# Understanding factual belief polarization: the role of trust, political sophistication, and affective polarization

**DOI:** 10.1057/s41269-022-00265-4

**Published:** 2022-10-20

**Authors:** Roderik Rekker, Eelco Harteveld

**Affiliations:** 1 Universiteit van Amsterdam: Afdeling Politicologie, Nieuwe Achtergracht 166, 1018 WV Amsterdam, The Netherlands; 2 University of Gothenburg, Göteborg, Sweden

**Keywords:** Factual belief polarization, Misperceptions, Affective polarization, Media trust, Climate change

## Abstract

Political opponents are often divided not only in their attitudes (i.e., ideological polarization) and their feelings toward each other (i.e., affective polarization), but also in their factual perceptions of reality (i.e., factual belief polarization). This paper describes factual belief polarization in the Netherlands around three core issues. Furthermore, this paper examines who are most susceptible to this type of polarization. Analyses on the 2021 Dutch Parliamentary Election Study reveal that citizens hold different perceptions than their political opponents about income inequality, immigration, and climate change. This type of polarization is strongest among citizens who have hostile feelings toward their political opponents and, paradoxically, among those who are highly educated and interested in politics. Trust in epistemic authorities did not mitigate factual belief polarization, perhaps because this trust has itself become politicized. These findings underline that factual belief polarization constitutes a core pillar of political polarization, alongside ideological and affective polarization.

## Introduction

In recent years, concerns over political polarization have grown among scholars and the general public alike. While the USA has seen growing divisions between the two major parties (Iyengar et al. 2012), Western Europe has been characterized by polarization around new cultural issues and populist radical right parties (Harteveld 2021). The literature on mass political polarization has commonly distinguished between ideological polarization (i.e., citizens’ overall divergence and partisan alignment in political views; Lelkes 2016) and affective polarization (i.e., citizens’ sympathy towards partisan in-groups and antagonism towards partisan out-groups; Iyengar et al. 2012). With rising controversies over issues such as climate change and COVID-19, it is however becoming increasingly clear that citizens are divided not only in their issue attitudes and their feelings toward each other, but also in their factual perceptions of reality. Indeed, about three-quarters of Americans believe that Republican and Democratic voters not only disagree over plans and policies, but also on “basic facts” (Pew Research Center 2019). This type of polarization has been labeled ‘factual belief polarization’ (Lee et al. 2021) and can be seen as a third core pillar of mass polarization alongside ideological and affective polarization (Rekker 2022).

Although an extensive literature exists on both misperceptions and polarization, there is a surprising lack of systematic research on factual belief polarization. As a result, little is known about what factual perceptions underlying citizens’ core political attitudes on issues such as economic redistribution or immigration. Instead, most research has focused on more extreme instances of misinformation and misperceptions such as conspiracy theories (e.g., Van Prooijen et al. 2015). The few studies that have examined factual perceptions regarding the most common political issues (e.g., Gorodzeisky and Semyonov 2020; Kuhn 2019) focused on only a single issue, without an overarching conceptual framework on polarization. Moreover, factual belief polarization is perhaps a rare instance of a topic on which there is a wealth of experimental and explanatory research (e.g., psychological studies on motivated reasoning; e.g., Kahan 2016), but a lack of observational and descriptive studies. Using data of the Dutch Parliamentary Election Study (DPES) of 2021, this study therefore provides an examination of what factual belief polarization looks like in the Netherlands. Specifically, we examine this type of polarization between citizens around three issues that are at the heart of political competition in Western Europe (e.g., Kriesi et al. 2008): economic redistribution, immigration, and climate change. These issues might plausibly be characterized by factual belief polarization because proponents of redistribution may perceive the highest level of income inequality, opponents of immigration may perceive the largest share of immigrants in the population, and opponents of climate policies may be most likely to question anthropogenic climate change. Moreover, we investigate who are most affected by such factual belief polarization. Citizens may be most likely to base their factual perceptions on their political attitudes when they lack trust in epistemic authorities such as science and the media (Rekker 2021). Paradoxically, people may also have a greater ability and motivation to bring their perceptions and attitudes in line when they are highly educated and interested in politics (Kahan et al. 2017).

The second aim of this study is to investigate the relation between factual belief polarization and affective polarization. Recent years have seen increasing levels of hostility between political opponents in the USA and many—though not all—societies in Western Europe (Boxell et al. 2022; Iyengar et al. 2019; Wagner 2021). Such affective polarization could be an important driver of factual belief polarization, as well as vice versa. When citizens have hostile feelings toward their political opponents, they may also be more likely to withdraw in an epistemic bubble. Conversely, people may be most prone to develop hostile feelings toward their political adversaries when they no longer share a common sense of reality. Research on this link has so far been sparse and inconclusive. Our study does not aim to establish the causal direction, but rather the strength of the potential association between factual belief polarization and affective polarization.

This examination constitutes a case study of the Netherlands in 2021. So far, most research on factual belief polarization has focused on the USA (Rekker 2021). It is therefore important to determine to what extent the same patterns can be found in West European multi-party systems, which are often (although not universally) characterized by lower levels of polarization (Wagner 2021). Like most other European countries, the Netherlands has however also seen a surge in science-skepticism among populist radical right parties (Gardiner 2019; Rekker 2021). This provides us with an interesting case to examine to what extent factual belief polarization has crossed the Atlantic.

## Theory and hypotheses

### Political polarization over factual beliefs

In one of the few attempts to systematically conceptualize and examine the phenomenon, Lee et al. (2021) introduced the term *factual belief polarization* for instances in which citizens have different factual perceptions than their political opponents. Such opponents can be citizens who support a different political party or people with different attitudes on a political issue. Factual belief polarization occurs when an objective fact is known according to evidence and expert opinion, but citizens’ factual perceptions are nonetheless correlated with their party preference or issue attitudes (Rekker 2022). In most cases, such factual beliefs (e.g., on climate science) have direct implications for contested political policies (e.g., reducing CO_2_ emissions).

Factual belief polarization is closely related to the concept of ‘misperceptions,’ which were defined by Nyhan and Reifler (2010, p. 305) as “cases in which people’s beliefs about factual matters are not supported by clear evidence and expert opinion.” Misperceptions have generally (though not consistently) been defined as incorrect beliefs that people hold with confidence (e.g., Flynn et al. 2017; Kuklinski et al. 2000). This separates misperceptions from ignorance, which is defined as lacking a correct belief on an issue. Despite the clear similarities, Rekker (2022) proposed two conceptual distinctions between misperceptions and factual belief polarization. First, misperceptions must be connected to citizens’ party preference or issue attitudes to qualify as *political* polarization, which excludes misperceptions about non-political matters such as the health risks of salty foods. Whereas misperceptions are about how citizens’ perceptions deviate from reality, factual belief polarization is about how these beliefs differ between political opponents. The second distinction is that factual belief polarization does not require that citizens hold their perceptions with a strong degree of confidence. For example, some people may have been misinformed about the number of immigrants in their country and confidently misperceive the actual number, whereas others may simply hold an incorrect assumption about the immigrant population. Only the former instance would constitute a misperception, but both examples constitute factual belief polarization as long as perceptions are correlated with citizens’ party preference or immigration attitudes. Because of the conceptual differences between both phenomena, misperceptions and factual belief polarization require a somewhat different research agenda. Studies on misperceptions have focused mostly on instances in which citizens have been misinformed and confidently hold blatantly inaccurate beliefs such as that Osama bin Laden is still alive. The research agenda for factual belief polarization, however, lies more in identifying the (often implicit) factual assumptions that are intertwined with citizens’ attitudes on the most central political issues.

Congruence between attitudes and issue positions may come about through two processes. First, factual belief polarization may emerge because citizens base their issue attitudes on their factual beliefs. Citizens who are not convinced that global warming is caused by human activity may for example oppose climate policies as a direct result of their factual perceptions. Although this causal relation seems almost self-evident, empirical findings on this effect are surprisingly inconclusive. Some experiments reveal that respondents change their attitudes when they are presented with factual information (Becker 2019; Cruces et al. 2013; Grigorieff et al. 2020; Scotto et al. 2017; Sides 2016), but other studies found no such effect (Hopkins et al. 2019; Jørgensen and Osmundsen 2020; Lawrence and Sides 2014; Trump 2018). Conversely, factual belief polarization may also arise because citizens’ attitudes guide their factual beliefs. People for example tend to process new information in a way that reinforces their attitudes, rather than challenging them (Nickerson 1998). Because of this *confirmation bias*, opponents of climate policies may for example pay particular attention to climate skeptics and avoid news about the impact of humans on global warming. Likewise, research shows that people’s attitudes on immigration importantly steer their interpretation of factual information on this issue (Glinitzer et al. 2021). Furthermore, uninformed citizens may use their political attitudes as a heuristic to fill the gaps in their knowledge (Herda 2013). Even when they have never been informed about a precise number, critics of immigration may for example reason that there ‘must be a lot of immigrants’ in the country because immigration otherwise could not be as big a problem as they perceive it to be.

Descriptive research on factual belief polarization around the most central political issues, such as redistribution and immigration, is surprisingly sparse. Instead, most studies have focused either on the psychological mechanisms that could explain factual belief polarization (e.g., Kahan 2016) or on more extreme forms of misperceptions such as conspiracy theories (e.g., Van Prooijen et al. 2015). Using data of the DPES of 2021, this study therefore provides a descriptive examination of what factual polarization looks like in the Netherlands. We examine factual belief polarization around three core political issues: economic redistribution, immigration, and climate change. For redistribution, a core factual perception is how people perceive the inequality between rich and poor. In one of the few attempts to examine such perceptions, the International Social Survey Programme (ISPP) included an item that asked respondents from 27 countries to estimate how much they think high-status (i.e., chairman of a large national company) and low-status (i.e., an unskilled factory worker) workers earn. Several studies on this survey have revealed that proponents of redistributive policies perceive a greater difference between the lowest and the highest incomes than opponents of redistribution (Bobzien 2020; García-Sánchez et al. 2020; Kuhn 2019). For immigration attitudes, a particularly important factual belief is how many immigrants people think are currently living in their country. Survey research in both the USA and Europe reveals that proponents and opponents of immigration are indeed divided in their factual perceptions of the immigrant population, with opponents perceiving the share of foreign-born citizens in their country to be larger in both absolute terms and relative to other countries (Gorodzeisky and Semyonov 2020; Herda 2013; Hjerm 2007; Nadeau et al. 1993; Semyonov et al. 2008; Sides and Citrin 2007).

By far the best documented instance of factual belief polarization is the dispute about anthropogenic climate change. The vast majority of studies on this controversy have focused on the US, where climate science has become highly politicized. In 2016, less than half (43%) of American citizens who identify as a Republican believed that climate change is caused by human activity, whereas the vast majority (84%) of Democrats accepted this scientific consensus (Dunlap et al. 2016). Although the politicization of climate science long seemed to be mostly an American phenomenon, this began to change after the Paris Agreement was signed in 2015. In the years that followed, many European populist radical right parties made climate skepticism into a core pillar of their ideology alongside Euroscepticism and immigration critique (Gardiner 2019; Rekker 2021). In the currently examined Dutch parliamentary elections of 2021, two radical right parties (i.e., the ‘Party for Freedom’ and ‘Forum for Democracy’) explicitly questioned the contribution of humans to global warming in their election manifesto. This politicization at the elite level is also starting to manifest itself in public opinion. In 2016 and 2017, Round 8 of the European Social Survey asked citizens from 20 European countries if they thought the climate is changing and whether this change is man-made. This survey revealed that citizens who politically identify with the Right are more likely to question that the climate is changing due to human activity than those who identify with the Left (Lübke 2021). Unsurprisingly, climate skepticism is not only associated with citizens’ partisan and ideological identity, but also with their issue attitudes on environmental policies (Whitmarsh 2011). Based on these previous studies on citizens’ perceptions of income inequality, the immigrant population, and climate change, we postulated our first hypothesis as follows:

#### Hypothesis 1

Citizens’ factual beliefs about income inequality are associated with their issue attitudes toward redistribution (H1a), beliefs about the immigrant population are associated with attitudes toward immigration (H1b), and beliefs about anthropogenic climate change are associated with attitudes toward climate policies (H1c).

### The role of trust in epistemic authorities

In modern democracies, epistemic authorities play a pivotal role in maintaining a shared reality. Institutions such as science and journalism have been bestowed with the task to provide the democratic debate with a factual basis. Trust in such epistemic authorities may therefore constitute a crucial barrier against factual belief polarization (Rekker 2021). As long as liberals and conservatives use and trust the same objective information sources, their factual beliefs should not become highly polarized. Citizens who distrust epistemic authorities may however be more likely to withdraw in their own epistemic bubble, for example by consuming ideological alternative media. Such ideological news media may fuel the proliferation of anti-scientific ideology and misperceptions, as well as the polarization of citizens’ worldviews more generally (Davis and Dunaway 2016; Garrett et al. 2019). Research has for example revealed that the use of conservative media diminishes citizens’ trust in science, which in turn decreases their belief in global warming, whereas the use of non-conservative media has the opposite effect (Hmielowski et al. 2014). In other words, citizens who distrust science and regular news media may be most likely to base their factual beliefs on their political attitudes rather than objective information. As long as citizens trust scientists and journalists, they may for example accept the scientific consensus on global warming regardless of their issue attitudes on climate policies. Our second hypothesis was therefore formulated as follows:

#### Hypothesis 2

Factual belief polarization is weaker among citizens who trust epistemic authorities such as science (H2a) and the media (H2b).

### The role of political sophistication

Whereas trust in epistemic authorities may mitigate factual belief polarization, there are other factors that may fuel it. Paradoxically, one of these factors is political sophistication, which we employ here to refer to the level of concern, familiarity and conceptualization citizens bring to their evaluation of politics (Neuman 1986). Although highly educated and politically interested citizens—which we take as imperfect but useful proxies of such sophistication—often hold more accurate beliefs than citizens with a lower level of political sophistication, this does not mean that they are less polarized. Well-informed government officials for example have more accurate factual beliefs than ordinary citizens, but on some issues their perceptions are nonetheless more aligned with their party preference (Lee et al. 2021). Likewise, highly educated citizens are less likely to reject the scientific consensus on climate change, but the correlation between political ideology and climate perceptions is nonetheless strongest among this group (Hamilton 2011; McCright and Dunlap 2011). Citizens who are highly educated and interested in politics may have a greater ability and motivation to reflect on their factual beliefs when forming an opinion on a political issue. Conversely, politically sophisticated citizens may also be most likely to bring their factual perceptions in line with their political attitudes through motivated reasoning (Kahan et al. 2017) or by taking cues from elites (e.g., Bakker et al. 2019; Zaller 1992). Our third hypothesis was therefore postulated as follows:

#### Hypothesis 3

Factual belief polarization is stronger among highly educated citizens (H3a) and among citizens who are interested in politics (H3b).

### The role of affective polarization

Only few studies (e.g., Lee et al. 2021) have examined political polarization from a perspective of misperceptions or vice versa. As a result, surprisingly little is known about the relation between factual belief polarization and other types of polarization. In particular, very few studies have examined the relation between factual belief polarization and affective polarization, which refers to citizens’ sympathy towards political in-groups and antagonism towards political out-groups (Iyengar et al. 2012; Wagner 2021). In the USA and several Western European societies, this type of polarization has increased since the beginning of the twenty first century, including in the Netherlands^1^ (Boxell et al. 2022; Dekker and Den Ridder 2019; Harteveld and Rekker 2021; Iyengar et al. 2012). There are good reasons to expect this development is connected to the rise of factual belief polarization during this same period.

On one hand, affective polarization may fuel factual belief polarization. People have a psychological need to form beliefs that maintain their status in an affinity group, which is commonly referred to as identity-protective cognition or politically motivated reasoning (Kahan 2016). When citizens become more emotionally invested in their political identity and more hostile toward opponents, they may develop a stronger tendency to exclusively trust identity-consistent information from in-group members, while disregarding identity-incongruent information from out-groups (Rekker 2022). In such instances, scientists may for example be distrusted when they are perceived as political opponents, while science skeptics are trusted when they are perceived as political allies. As Roberts (2020) eloquently put it: “Tribal epistemology happens when tribal interests subsume transpartisan epistemological principles, like standards of evidence, internal coherence, and defeasibility. ‘Good for our tribe’ becomes the primary determinant of what is true; ‘part of our tribe’ becomes the primary determinant of who to trust.” Illustrating the relevance of affective polarization for factual belief polarization, a panel study on American voters revealed that the use of partisan media fuels misperceptions and that this relation is partly mediated by increased levels of affective polarization (Garrett et al. 2019). Moreover, Americans who hate their political opponents are more likely to share political fake news on Twitter (Osmundsen et al. 2020). The most direct support for a causal effect of affective polarization on factual beliefs comes from an experiment, which revealed that experimentally induced affective polarization increased Democrats’ perception of the level of unemployment under Donald Trump’s presidency (Broockman et al. 2020). This effect should however be interpreted with caution because it was not found consistently in all instances.

Conversely, factual belief polarization could also be a source of affective polarization. Although empirical research on this effect is currently lacking, it stands to reason that a shared sense of reality can constitute a barrier against political hostility by ensuring at least a basic level of understanding for the other’s position (Rekker 2022). If factual beliefs grow apart, political camps may however start to see the other not only as having the wrong ideas, but as not getting reality at all, which is easier to condemn than mere disagreement. Opponents of climate policies may for example maintain some empathy for climate activists as long as they at least share their factual belief that global warming is man-made. Skeptics who are convinced that climate change is a hoax may however be much more hostile toward the climate movement, as well as vice versa.

Because the present study draws from cross-sectional survey data, we cannot distinguish between both potential directions of causality between factual belief polarization and affective polarization. This study does however provide a descriptive examination of the association between both types of polarization regarding some of the most central issue attitudes in West European politics. As of yet, no study has established this association. Based on the theoretical plausibility of a bidirectional causal effect between political hostility and factual perceptions, we formulated our fourth hypothesis as follows:

#### Hypothesis 4

Factual belief polarization is stronger among citizens with hostile feelings toward their political opponents.

## Method

### Data

The DPES 2021 (Jacobs et al. 2021) was collected before and after the legislative elections of March 17. The items used in this study were included in the post-election survey. The online survey was partly based on a fresh probability sample of 6600 Dutch voters, of which 2400 participated. The rest of the sample was recruited from the existing LISS-Panel (Longitudinal Internet Studies for the Social Sciences) of which 2797 respondents were approached and 2137 participated in the post-election study.

### Measures

Respondents’ *factual beliefs* were measured with items that asked them about their perceptions regarding redistribution, immigration, and climate change. For redistribution, this item asked respondents “How many times more do you think the 10% households with the highest incomes earn compared to the 10% households with the lowest incomes? This is about the amount of money that a family can spend after taxes (disposable household income).” The response scale ranged from 1× to 15×. Based on data from the ‘Organisation for Economic Co-operation and Development’ (OECD 2016), the true value of this indicator is ‘7×.’ For factual beliefs on immigration, the item was phrased as follows: “What do you think is currently the share of immigrants in the Netherlands? By immigrants, we mean people who were born in another country (first generation immigrants) as well as their children (second generation immigrants).” The response scale ranged from 0 to 100%. Based on census data, Statistics Netherlands (CBS 2021) reports that the true share of first- and second-generation immigrants is 25% of the Dutch population. To measure respondents’ factual beliefs about climate change, they were asked “How convinced are you that climate change is mainly caused by human activity? Please place yourself on a scale from 0 to 100% where 0% means that you think it is extremely unlikely that climate change is caused mainly by human activity, 100% means that you are sure that climate change is caused mainly by human activity, and 50% means that you are unsure whether or not climate change is caused mainly by human activity.” The response scale ranged from 0 to 100%. Based on reports from the Intergovernmental Panel on Climate Change (IPCC 2013, 2021) any answer between about 95% (the estimate from the 2013 IPCC report) and 100% confidence (the 2021 IPCC report describes the evidence for anthropogenic global warming as ‘unequivocal’) can be considered in line with the scientific consensus. Another study on factual belief polarization revealed that the vast majority of respondents (i.e., 91.2% in an American sample) have a sufficient understanding of ratios and percentages to answer this type of questions (ANONYMIZED).

Respondents’ *issue attitudes* on redistribution were measured with an item that asked them: “Where would you place yourself on a line from 1 to 7, where 1 means differences in income should be increased and 7 means that differences in income should be decreased?” For immigration, respondents were presented with the statement “There are too many people of foreign origin or descent in the Netherlands,” with response categories ranging from 1 (fully agree) to 7 (fully disagree). This immigration item was presented only to the 2137 respondents who were part of the LISS-Panel. For climate change, the question read “How would you place yourself on a line from 1 to 7, where 1 means that the measures against climate change have gone too far and 7 that the measures should go further?”

Respondents’ *trust in epistemic authorities* was measured as part of a larger battery on institutional trust. Among other institutions, respondents were asked to indicate to what extent they trust ‘science’ and ‘the press’ (which we take as a proxy for trust in the media more generally) on a scale ranging from 1 (very much) to 4 (not at all). Respondents’ *political sophistication* is operationalized as their educational attainment and their level of interest in politics. We coded education as a dichotomy that distinguishes respondents with either a low or a middle educational level (primary, secondary, and lower vocational tertiary education) from those with a high educational level (higher vocational tertiary education or university). Respondents reported their political interest as ‘not,’ ‘fairly,’ or ‘very’ interested.

*Affective polarization* is operationalized as dislike towards issue-based outgroups (Hobolt et al. 2021) on each of the three topics. This means that we only capture the outgroup derogation component of affective polarization and not ingroup favoritism, which is often included in the operationalization of affective polarization (see Iyengar et al. 2019) but not available in the data. However, we note that our strategy likely leads to a conservative estimate of the total effect of affective polarization: ingroup attachment might well further foster factual belief polarization. Regardless, most of the normative concerns have been directed at the rise of specifically negative bias, and our study allows to investigate whether this in turn is associated with more factual belief polarization. Following the wording of the American National Election Survey (ANES), respondents rated people who disagree with them on each of the issues on a so-called feeling thermometer ranging from 0 (cold) to 100 (warm). Those opponents were depicted as holding the oppositive view from the respondent. For instance, respondents who indicated to be in favor of more measures against climate change had to evaluate ‘People who think the government’s measures against climate change have gone too far’ and respondents who favor smaller income differences evaluated ‘People who think income differences should be bigger.’ Respondents who gave a neutral response were provided an outgroup at random. For a small number of respondents (3.2%) who completed the questionnaire on paper, randomization and automatic routing were not available. These respondents were therefore asked to provide a temperature for “People who think different than you about the question whether [description of the issue].” The colder the thermometer rating, the higher the respondent is deemed to be affectively polarized towards an issue outgroup. To facilitate the interpretation of the results in our tables, we recoded affective polarization as 100 minus the thermometer score for all regression models.

The DPES did not contain an affective polarization item about immigration. Instead, we use a thermometer item about integration, in which respondents evaluated opponents on the question whether immigrants can ‘keep their own culture’ or should ‘adjust to Dutch culture.’ As part of the nativism-cosmopolitanism issue dimension, this item correlates significantly with the item on immigration, but imperfectly so, at *r* = 0.48. Because the routing for this particular thermometer item was based on respondents’ integration attitude, we rely on the integration rather than immigration attitude in the model in which we test the impact of affective polarization (Model 4). All other models use the item on immigration. “Appendix” presents the descriptive statistics of the variables under study.

### Strategy of analysis

We test our hypotheses using a set of regression models explaining factual beliefs regarding each of the three issues. To study the presence of factual belief polarization (H1), we first predict each factual belief by respondents’ attitudes on the respective issue (Model 1). As conceptualized in the theoretical framework, we take a relation between the two to be evidence of factual belief polarization. To study which factors strengthen or weaken factual belief polarization, we subsequently estimate separate models interacting attitudes with the moderators identified in our hypotheses (Models 2–4): trust in media (H2a) and science (H2b), education (H3a), political interest (H3b) and affective polarization (H4). In a penultimate model we include all these interactions simultaneously (Model 5). To assess the role of possible confounders, the last model (Model 6) in addition includes socio-demographic controls for age, sex, immigrant background (first- or second-generation), self-reported social class, and labor market position (in a paid job versus not in a paid job). All models contain heteroscedasticity-robust standard errors as well as political and demographic sample weights. Because we have directional hypotheses, we report significance based on one-sided *t*-tests.

## Analyses and results

Table 1 presents all regression models. We discuss each hypothesis in turn based on the main effect (Model 1) and the models containing one interaction (Models 2 to 4). We report in the text whenever the results differ in a model with simultaneously estimated interactions (Model 5) and/or control variables (Model 6). We visualize the results to provide a better understanding of the effect size and of the direction of interactions. In each figure, the vertical axis ranges from the 5th to the 95th percentile of respondents’ factual beliefs, hence providing some measure of comparability between the different dependent variable scales. For easier reference, figures visualizing an interaction effect also report the *p*-value of the interaction term (Models 2–4).

**Table 1:** Regression models

	Redistribution								Immigration			
	Model 1	Model 2a	Model 2b	Model 3a	Model 3b	Model 4	Model 5	Model 6	Model 1	Model 2a	Model 2b	Model 3a
	*b*/se	*b*/se	*b*/se	*b*/se	*b*/se	*b*/se	*b*/se	*b*/se	*b*/se	*b*/se	*b*/se	*b*/se
Attitude	0.249**	− 0.001	0.379	− 0.216	− 0.067	− 0.273	− 0.873	− 0.930	2.222***	0.723	1.346	4.087*
	(0.089)	(0.284)	(0.434)	(0.279)	(0.336)	(0.303)	(0.567)	(0.573)	(0.511)	(1.345)	(2.085)	(1.919)
Trust in the press		− 0.197					− 0.286	− 0.094		− 3.866*		
		(0.595)					(0.706)	(0.704)		(1.773)		
Attitude # Trust in press		0.104					0.093	0.034		0.545		
		(0.118)					(0.135)	(0.134)		(0.574)		
Trust in science			0.529				0.556	0.662			− 4.394*	
			(0.602)				(0.727)	(0.739)			(2.213)	
Attitude # Trust in science			− 0.045				− 0.136	− 0.139			0.064	
			(0.130)				(0.153)	(0.152)			(0.675)	
High educated				− 0.990			− 0.893	− 0.865				1.614
				(0.871)			(0.903)	(0.954)				(3.684)
Attitude # High educated				0.323*			0.218	0.257				− 1.644
				(0.171)			(0.173)	(0.180)				(1.152)
Political interest					− 0.254		− 0.489	− 0.795				
					(0.726)		(0.712)	(0.733)				
Attitude # Political interest					0.167		0.201	0.270*				
					(0.149)		(0.143)	(0.145)				
Affective polarization						− 0.024	− 0.027	− 0.025				
						(0.016)	(0.017)	(0.018)				
Attitude # Affective polarization					0.006*	0.007*	0.007*					
						(0.004)	(0.003)	(0.003)				
Age at election day								0.025**				
								(0.009)				
Female								0.027				
								(0.244)				
Immigration background								− 0.800**				
								(0.335)				
Class (ref: Upper class)												
Upper middle class								0.160				
								(0.506)				
Middle class								0.840*				
								(0.507)				
Upper working class								1.050*				
								(0.583)				
Working class								1.064				
								(0.667)				
Has paid job								0.733*				
								(0.337)				
Intercept	9.87***	10.36***	8.286***	11.29***	10.34***	11.80***	13.46***	11.18***	26.25***	35.64***	41.93***	24.75***
	(0.431)	(1.393)	(2.030)	(1.426)	(1.612)	(1.261)	(2.769)	(3.091)	(1.636)	(4.253)	(6.968)	(6.582)
*N*	2580	2527	2526	2421	2452	2499	2267	2184	1715	1678	1667	1547
*R*^2^	0.004	0.007	0.005	0.012	0.010	0.007	0.024	0.045	0.027	0.041	0.062	0.039
Adjusted *R*^2^	0.004	0.006	0.004	0.011	0.008	0.006	0.019	0.037	0.027	0.039	0.061	0.037

	Immigration				Climate							
	Model 3b	Model 4	Model 5	Model 6	Model 1	Model 2a	Model 2b	Model 3a	Model 3b	Model 4	Model 5	Model 6
	*b*/se	*b*/se	*b*/se	*b*/se	*b*/se	*b*/se	*b*/se	*b*/se	*b*/se	*b*/se	*b*/se	*b*/se
Attitude	3.410*	− 1.460	1.936	3.425	9.028***	10.205***	7.193***	5.522***	3.805***	0.050	− 3.586*	− 2.381
	(2.055)	(0.934)	(2.816)	(2.711)	(0.310)	(1.032)	(1.513)	(1.032)	(1.112)	(1.214)	(2.055)	(2.026)
Trust in the press			− 3.040	− 1.191		5.996**					6.565***	7.325***
			(1.893)	(1.864)		(2.226)					(2.069)	(1.925)
Attitude # Trust in press			0.723	0.440		− 0.675					− 1.003**	− 0.983**
			(0.592)	(0.580)		(0.417)					(0.385)	(0.356)
Trust in science			− 0.691	− 0.882			2.195				0.612	1.761
			(2.694)	(2.454)			(2.393)				(2.243)	(2.285)
Attitude # Trust in science			− 0.407	− 0.239			0.458				0.533	0.170
			(0.781)	(0.715)			(0.453)				(0.433)	(0.440)
High educated			1.934	2.239				− 8.752**			− 2.522	− 2.750
			(3.228)	(3.061)				(3.226)			(2.885)	(2.867)
Attitude # High educated			− 1.253	− 1.331				2.337***			0.962*	0.794
			(1.012)	(0.969)				(0.613)			(0.526)	(0.519)
Political interest	− 1.878		− 3.179	− 1.946					− 10.773***	− 7.630**	− 6.070**	
	(3.001)		(2.966)	(2.577)					(2.471)		(2.486)	(2.328)
Attitude # Political interest	− 0.899		− 0.020	− 0.142					2.581***		1.649***	1.325**
	(0.885)		(0.865)	(0.776)					(0.484)		(0.473)	(0.433)
Affective polarization		− 0.125*	− 0.101	− 0.049						− 0.573***	− 0.513***	− 0.577***
		(0.060)	(0.067)	(0.060)						(0.076)	(0.087)	(0.080)
Attitude # Affective polarization	0.022*	0.010	0.004						0.119***	0.103***	0.113***	
		(0.012)	(0.020)	(0.019)						(0.015)	(0.017)	(0.016)
Age at election day				− 0.151***								− 0.122***
				(0.046)								(0.031)
Female				2.324*								− 1.583*
				(1.004)								(0.959)
Immigration background				5.841***								0.358
				(1.530)								(1.342)
Class (ref: Upper class)												
Upper middle class				− 2.601								− 1.947
				(3.494)								(2.055)
Middle class				− 0.876								− 3.575*
				(3.604)								(2.101)
Upper working class				− 1.026								− 4.544
				(3.960)								(2.833)
Working class				1.177								− 1.464
				(4.141)								(2.809)
Has paid job				− 1.767								− 1.189
				(1.597)								(1.173)
Intercept	30.85***	37.52***	46.98***	42.88***	28.11***	16.280**	22.694**	41.25***	49.97***	70.95***	71.71***	79.03***
	(7.031)	(4.457)	(9.586)	(10.592)	(1.646)	(5.355)	(7.691)	(5.230)	(5.534)	(5.858)	(10.000)	(10.407)
*N*	1572	2884	1459	1380	3301	3207	3198	3076	3124	3137	2796	2668
*R*^2^	0.069	0.006	0.087	0.157	0.376	0.394	0.397	0.376	0.381	0.421	0.453	0.482
Adjusted *R*^2^	0.068	0.005	0.080	0.145	0.376	0.394	0.397	0.375	0.380	0.420	0.451	0.478

***p < 0.001; **p < 0.01; *p < 0.05 (one-sided test)

### Political polarization over factual beliefs

**Fig. 1:**
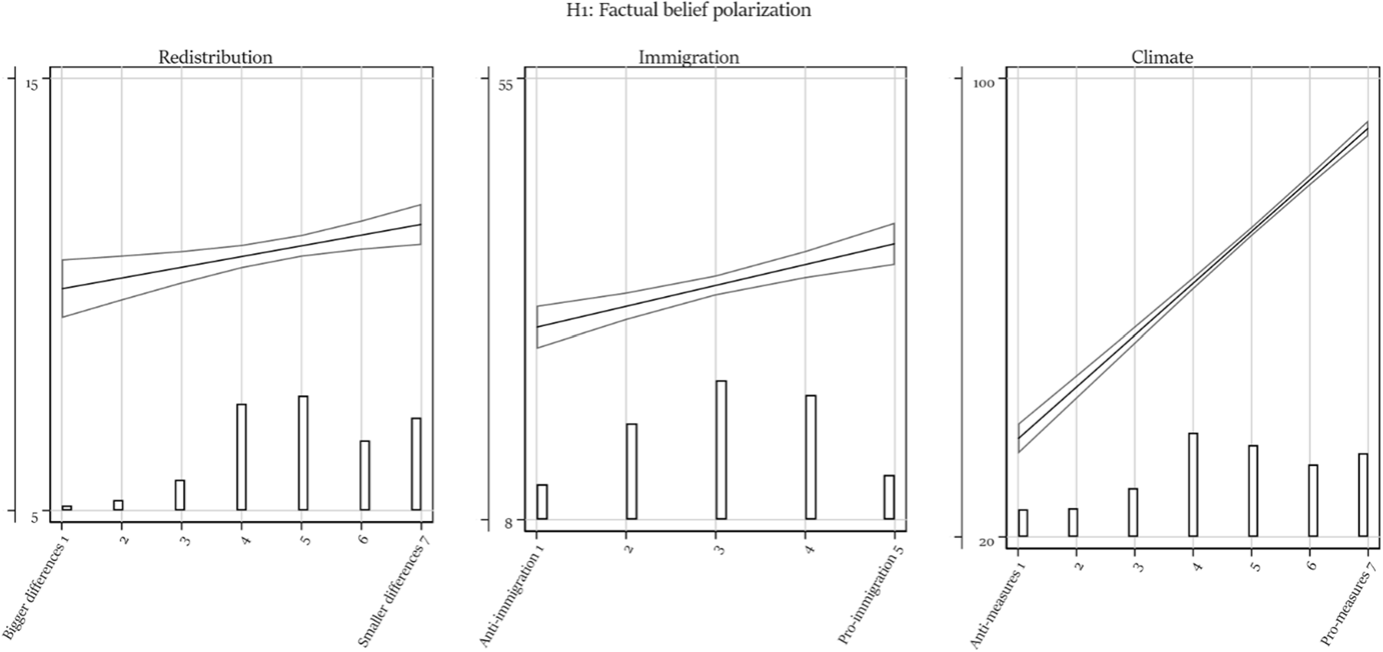
The relation between factual beliefs and issue attitudes. Predicted values with 95% confidence interval. With overlaid distribution of attitudes. Based on Model 1 of Table 1

Were the Dutch polarized over facts during the 2021 parliamentary elections? We find evidence for factual belief polarization around all three examined issues (Fig. 1). Citizens who support redistribution perceive larger income differences and those who oppose immigration perceive a larger immigrant population. Finally, and most starkly, those who are in favor of measures against climate change are much more convinced that global warming is man-made. The magnitude of factual belief polarization is very substantial around this issue: Those who are least open to climate measures assign a 37% likelihood that climate change is man-made, against 91% among those most supportive of climate measures. Inspection of the explained variance confirms this. Climate attitudes explain more than 36% (*r* = 0.61) of the variance in factual beliefs about climate change, whereas the respective *R*^2^ is 2.7% in the case of immigration and less than one percent in the case of redistribution.

### The role of trust in epistemic authorities

**Fig. 2:**
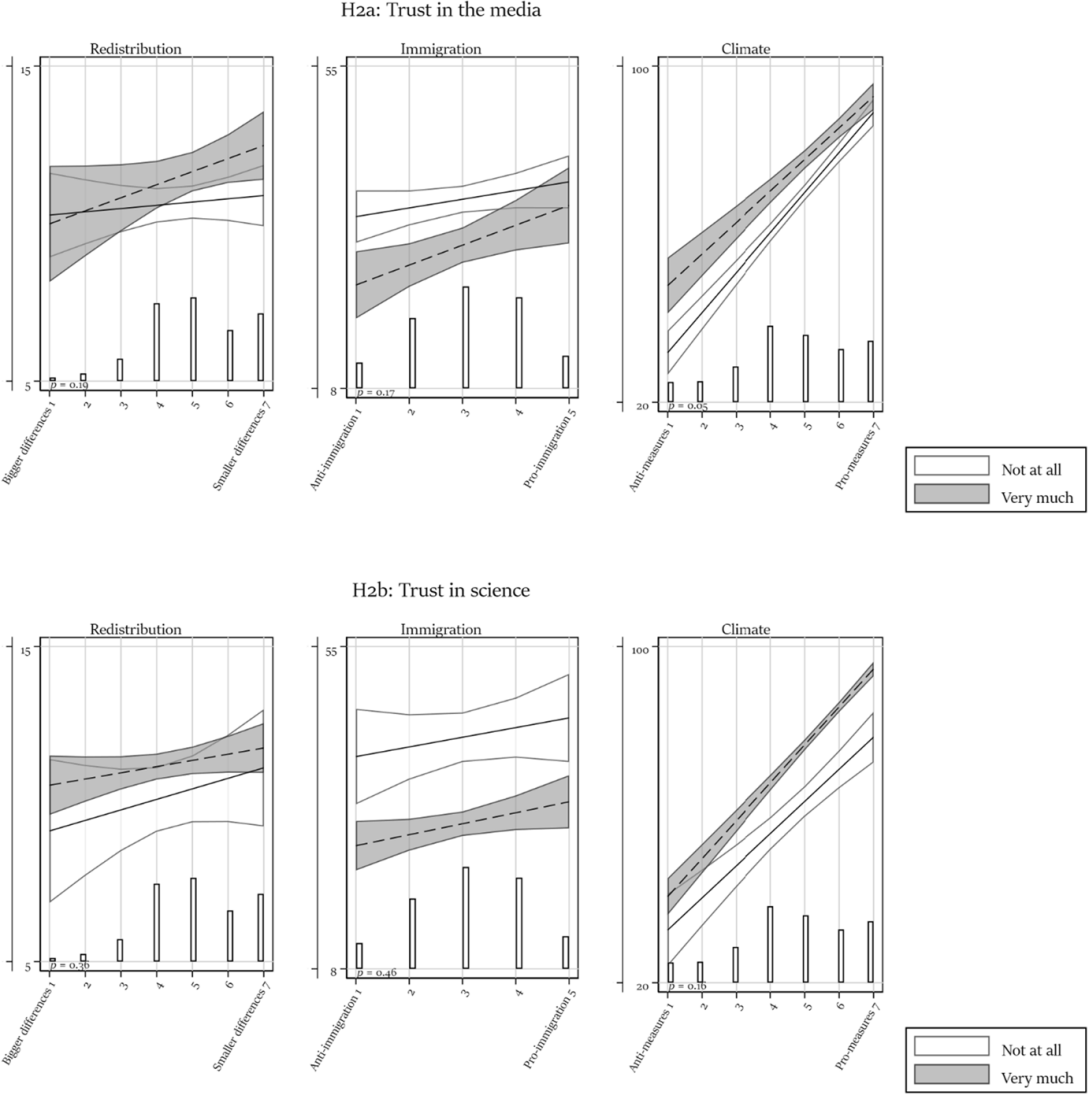
Moderation by trust in epistemic authorities. Predicted values with 95% confidence interval. Based on Models 2a and 2b in Table 1. With overlaid distribution of attitudes. The *p*-value relates to the interaction term and is based on a one-sided test. Does not include the intermediate values of political interest

Does citizens’ trust in the media or science mitigate factual belief polarization? Based on Models 2a and 2b, Fig. 2 visualizes the interaction between trust in these institutions and attitudes. While trust in media and science has an important main effect, few of the interaction terms are significant. One possible exception is trust in the media in the context of the issue of climate, which significantly interacts with attitudes in the full model (Model 5). Even then, the effect is not very substantial. In other words, except for possibly the issue of climate, trust in epistemic authorities does not dampen factual belief polarization. At least, not regarding the issues investigated in our study.

### The role of political sophistication

**Fig. 3:**
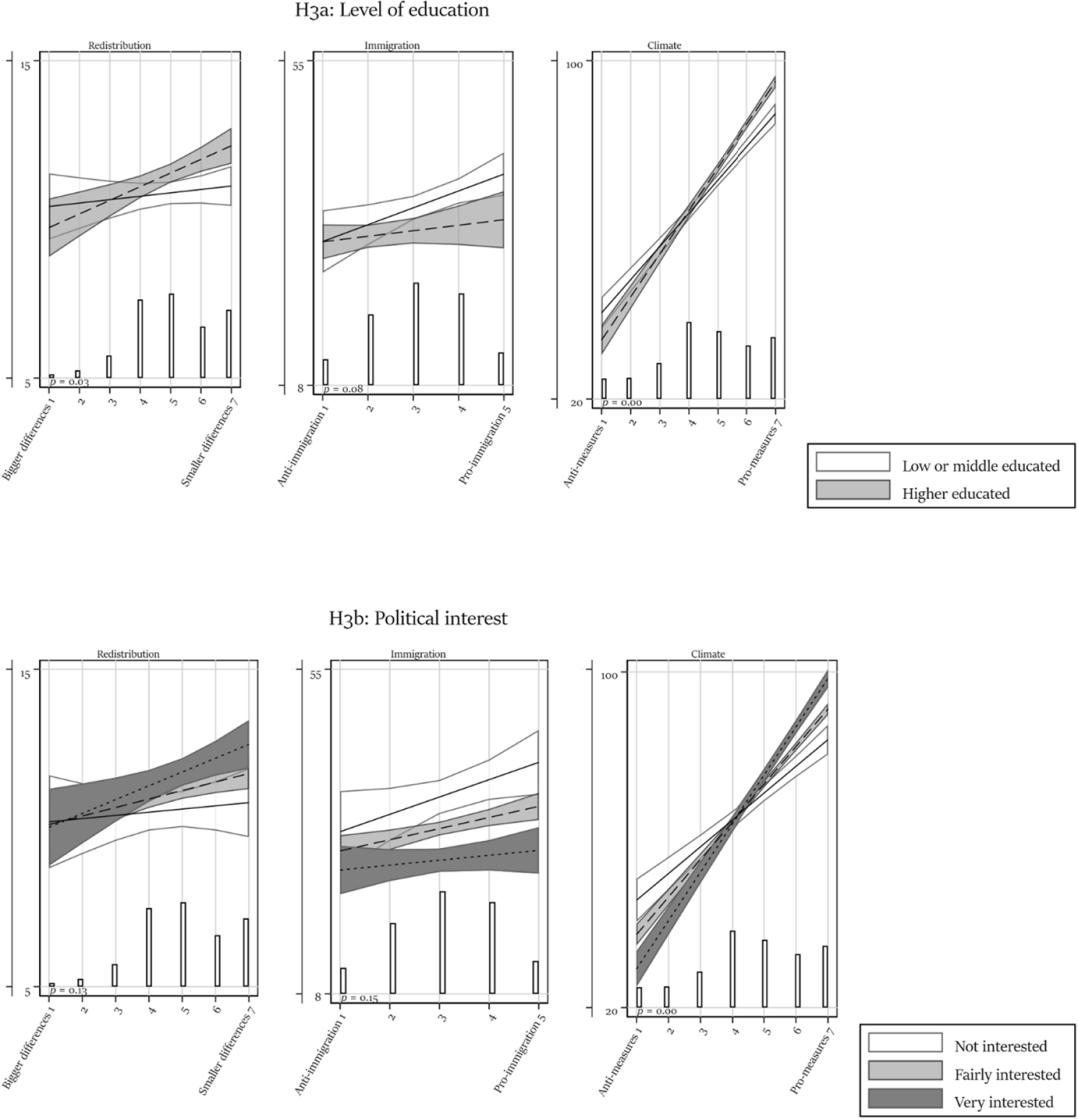
Moderation by political sophistication. Predicted values with 95% confidence interval. Based on Models 2a and 2b in Table 1. With overlaid distribution of attitudes. The *p*-value relates to the interaction term and is based on a one-sided test, except for the issue of immigration where the effects are opposite from expected

The second set of potential moderators relate to political sophistication. Figure 3 presents evidence that having a higher level of education is associated with more factual belief polarization around redistribution and climate change (it should be noted that in the case of redistribution the effect is not significant in a two-sided test). In the case of immigration, no significant interaction exists and the pattern is descriptively opposite from expected. Political interest heightens factual belief polarization over climate, but there is no robust evidence that it also does so over redistribution and immigration. All in all, we find some evidence that factual belief polarization is strongest among those who are most committed to politics, but its impact differs between issues.

### The role of affective polarization

**Fig. 4:**
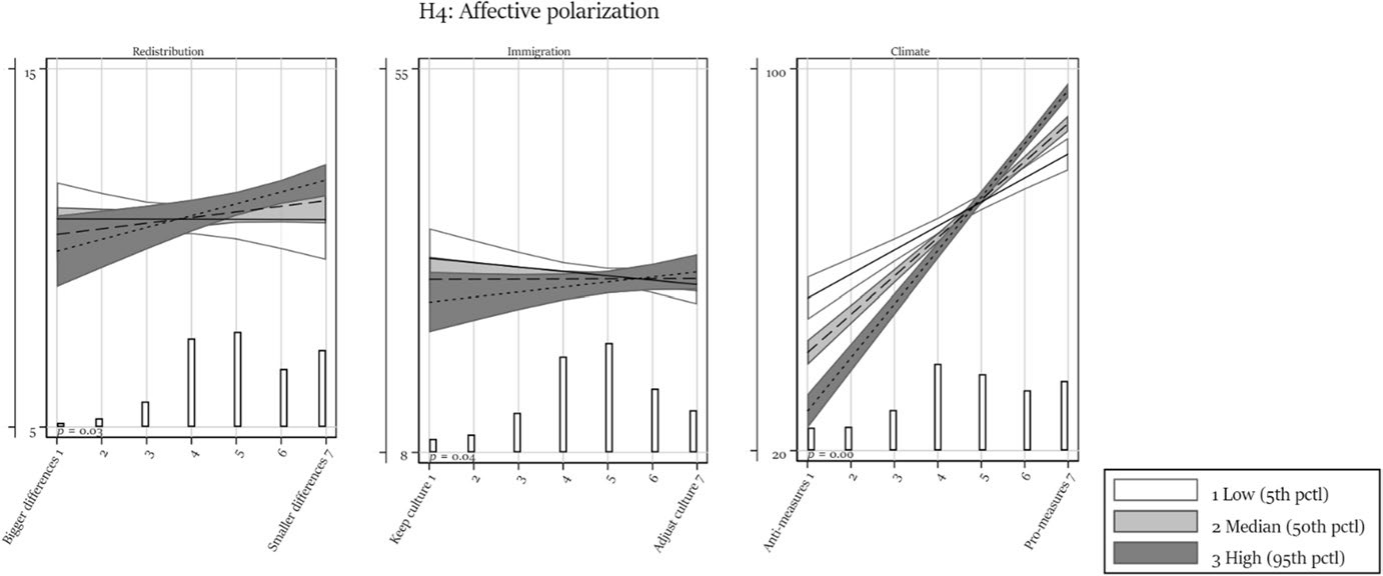
Moderation by affective polarization. Predicted values with 95% confidence interval. Based on Models 2a and 2b in Table 1. With overlaid distribution of attitudes. The *p*-value relates to the interaction term and is based on a one-sided test. The different degrees of affective polarization (a continuous measure) are based on feeling thermometer percentiles: 58 degrees (5th percentile), 33 degrees (50th percentile), and 6 degrees (95th percentile)

The final moderator is affective polarization. As Fig. 4 shows, affective polarization is an important predictor of factual belief polarization with substantial interactions in all three cases (although it should be noted that the interaction is only significant in a one-sided test in the case of redistribution and immigration). Those citizens who are most affectively polarized are more (or in the case of climate change much more) likely to have factual beliefs that are congruent with their attitudes. As noted above, we cannot establish whether the affective distance is cause or consequence of this alignment of facts and attitudes, but the substantive effects testify that either or both mechanisms are at work.

## Discussion

This study examined what factual belief polarization looks like in the Netherlands. We found that Dutch citizens have different factual beliefs than their political opponents on three core political issues: proponents of redistribution assume higher levels of inequality, critics of immigration perceive a larger immigrant population, and advocates of climate policies are more certain that global warming is man-made. The magnitude of factual belief polarization however differs between the three examined issues. The weakest link between attitudes and perceptions was found for redistribution. A possible explanation for this finding is that the redistribution issue has been characterized by decreasing levels of overall polarization in the Netherlands since the 1990s (Dekker and Den Ridder 2019). Because redistribution is more about people’s interests and less about their identity, the issue may also be less susceptible to the kind of psychological processes that drive affective polarization and factual belief polarization (Gidron et al. 2020; Harteveld 2021). By far the strongest factual belief polarization was found for climate change. Much more than for the other two issues, citizens seem to base their opinion about climate policies on their factual beliefs or vice versa. In fact, the correlation between attitudes and perceptions is so strong for this issue (*r* = 0.61) that only few Dutch citizens combine outspoken skepticism about climate policies with certainty about anthropogenic global warming.

The goal of this study was to examine factual belief polarization, which distinguishes this type of research from studies on misperceptions (e.g., Flynn et al. 2017). For factual belief polarization, it matters not so much how citizens’ perceptions differ from an objective reality, but rather how these beliefs differ from those of their political opponents as indicated by an association with party preference or issue attitudes. Nonetheless, our analyses can also shed some light on the accuracy of citizens’ factual perceptions. With regard to the income distribution, it appears that Dutch voters on average overestimate the difference between the lowest and the highest incomes (mean answer: 10.9×; median answer: 10×; true value: 6.8×; OECD 2016). This finding is remarkable given that previous research revealed that people tend to underestimate the level of inequality in their country (e.g., Kiatpongsan and Norton 2014). A possible explanation for this contradiction is that inequality is relatively easy to overestimate in the Netherlands because it has a relatively egalitarian income distribution (OECD 2016). Alternatively, respondents may have been steered to overestimation by the response scale which ran from 1× to 15×. On the issue of immigration, our findings are in line with previous research (e.g., Gorodzeisky and Semyonov 2020) in revealing that citizens on average overestimate the number of immigrants in their country (mean answer: 29.6%; median answer: 28%; true value: 25%; CBS 2021). Our analyses however also reaffirm a finding from previous research (Herda 2013) that overestimation is far from universal: 53% of respondents in our study overestimated the size of the immigrant population, 7% knew the precise number, and 40% underestimated the true size. Indeed, the median voter of pro-immigration parties (e.g., Volt, GroenLinks, D66, PvdA) in this dataset underestimates the size of the immigrant population (Harteveld and Rekker 2021). With regard to climate change, Dutch voters on average appear to be less certain than scientists that global warming is man-made (mean answer: 68.6%; median answer: 75%; scientific consensus: 95–100%; IPCC 2013, 2021).

Our hypothesis that trust in epistemic authorities would mitigate factual belief polarization was not confirmed by the data. Instead, the correlation between attitudes and factual perceptions turned out to be about equally strong among citizens with low and high levels of trust in science and the media. For factual beliefs about income inequality and immigration, this null-result might suggest that citizens’ (mis)perceptions do not originate from (dis)trust in epistemic authorities or from misinformation from alternative media. Instead, people’s factual beliefs on these issues may be implicit assumptions that they formed with their issue attitudes as a heuristic (Rekker 2022). This explanation however seems less plausible for factual beliefs on climate change, for which particularly trust in science is directly relevant. We reasoned that citizens who trust science would generally endorse the scientific consensus on global warming even if they oppose climate policies. A possible explanation for the absence of such an interaction effect is that there were simply not enough respondents who trust science but simultaneously oppose climate policies. Indeed, trust in science has itself become increasingly associated with political attitudes in both the USA (Gauchat 2012) and the Netherlands (Harteveld and Rekker 2021). In our sample, the correlation between issue attitudes on climate policies and trust in science was *r* = 0.30. Because of this politicization of trust, citizens’ trust in epistemic authorities may be a mediator of the association between attitudes and factual beliefs rather than a moderator as we hypothesized in this study. Another explanation for the null-results in our study is that citizens who trust science engage in motivated reasoning to interpret the scientific consensus to be more in line with their attitudes. Further research may shed more light on this issue. The null-results in the present study do however suggest that high levels of trust in science and the media cannot always prevent factual belief polarization.

Our results partially supported our hypothesis that factual belief polarization would be stronger for citizens with a higher level of political sophistication. In line with previous research on climate change (Hamilton 2011; McCright and Dunlap 2011), our results unambiguously revealed that highly educated and politically interested citizens are more polarized in their factual perceptions of global warming. Our results however failed to confirm that these findings generalize to other issues besides climate change. Although we found a similar moderating effect of educational level for factual belief polarization about income inequality, the interaction effect with political interest for the same issue did not reach statistical significance. Furthermore, neither educational level nor political interest moderated the association between issue attitudes and factual beliefs on immigration. On immigration and climate change (but not income inequality) highly educated and politically interested respondents furthermore provided more accurate answers than less politically sophisticated citizens. Across the three issues, the general pattern of our findings is therefore consistent with previous studies that demonstrated how politically sophisticated people can simultaneously be more accurate and more polarized in their factual beliefs (Lee et al. 2021). This has ambiguous implications for efforts to reduce misperceptions by stimulating citizens’ engagement and education: while increasing citizens’ cognitive engagement with issues might help them to paint a more accurate perception, the increased sophistication might nevertheless result in further factual belief polarization. Furthermore, such differential patterns for misperceptions and factual belief polarization emphasize the importance of making a conceptual distinction between both phenomena (Rekker 2022).

For all three examined issues, the results confirmed our hypothesis that factual belief polarization would be stronger among citizens with hostile feelings toward their political opponents. This pattern is consistent with a causal effect in either or both directions between the two types of polarization. On one hand, political hostility may drive people in their own epistemic bubbles where they exclusively trust attitude-congruent information from political allies. Conversely, citizens who no longer share a common sense of reality with their political opponents may be most likely to lose any understanding for their position or even sympathy for them as human beings. Our findings suggest that such a spiral of polarization may currently be taking place especially around the issue of climate change. Compared to redistribution and immigration, the climate change issue is characterized by higher levels of factual belief polarization (Fig. 1), higher levels of affective polarization (based on the same dataset; Harteveld and Rekker 2021), and a stronger connection between both types of polarization (Fig. 4). This issue may be particularly susceptible to different types of polarization because proponents of climate policies view global warming as an existential threat to the planet, while skeptics view such policies as an existential threat to their way of life. If so, the (likely) increase in the coming years of the salience of the climate issue might result in more polarized societies. Interestingly, the finding that factual belief polarization and affective polarization often come together may also explain why some issues are characterized by stronger levels of factual belief polarization than others. For example, the lower level of factual belief polarization around redistribution in this study may be tied to the lower levels of affective polarization around this issue (Harteveld 2021; but cf. Iyengar et al. 2012).

A limitation of this study is that it provides a case study of a single country. Most studies on factual belief polarization (as well as affective polarization) have so far focused on the USA (Rekker 2021). Our findings provide clear evidence that factual belief polarization around core issues, including the politicization of climate science, also exists in the Netherlands. The susceptibility of the climate issue for all three types of polarization is likely similar in other (West European) contexts. For other issues, the level of factual belief polarization might differ more starkly between contexts, depending—among other things—on issue salience and elite polarization. There is no a priori reason, however, to expect that in other societies there will exist very different associations between factual belief polarization on the one hand and political sophistication and affective polarization on the other. Still, future research should determine to what extent the same patterns can be found in other West European countries. A second shortcoming of our study is that its cross-sectional design is not suitable for causal inferences. Experimental studies are needed to determine the direction of causality between factual belief polarization and affective polarization. Finally, it should be noted that some findings in our study were only significant using one-sided hypothesis testing. If future studies can replicate our findings in other contexts, this will also provide further evidence for their statistical robustness.

To conclude, this study demonstrated that factual belief polarization exists around three core political issues in Dutch politics and that it is connected to other types of polarization. These findings underline that factual belief polarization constitutes a core pillar of political polarization, alongside ideological and affective polarization. Particularly the issue of climate change could be subject to a toxic spiral of these three types of polarization. This alarming possibility warrants more research, since climate change could well become an even more salient political issue in the foreseeable future.

## Appendix: Descriptive statistics

**Table:**

Variable	*N*	Mean or proportion	Standard deviation	Min	Max
Immigration					
Attitude^a^	1890	3.11	1.10	1	5
Alternative attitude^b^	3640	4.65	1.46	1	7
Factual perception^c^	2995	27.53	14.13	2	100
Outgroup sympathy	3287	40.59	24.22	0	100
Redistribution					
Attitude^d^	3537	5.08	1.40	1	7
Factual perception^e^	2624	11.38	5.45	1	153
Outgroup sympathy	3216	29.64	23.70	0	100
Climate					
Attitude^f^	3552	4.65	1.72	1	7
Factual perception^g^	3367	70.68	25.27	0	100
Outgroup sympathy	3248	31.39	21.31	0	100
Trust in epistemic authorities					
Trust in the press	3526	2.38	0.77	1	4
Trust in science	3508	3.25	0.67	1	4
Sophistication					
Low or middle education	4338	0.56	0.50	0	1
Higher education	4338	0.44	0.50	0	1
Political interest	4474	1.95	0.60	1	3

^a^There are too many people of foreign origin or descent in the Netherlands

^b^Do you think immigrants can ‘keep their own culture’ or should ‘adjust to Dutch culture’?

^c^What do you think is currently the share of immigrants in the Netherlands? By immigrants, we mean people who were born in another country (first generation immigrants) as well as their children (second generation immigrants)

^d^Where would you place yourself on a line from 1 to 7, where 1 means differences in income should be increased and 7 means that differences in income should be decreased?

^e^How many times more do you think the 10% households with the highest incomes earn compared to the 10% households with the lowest incomes? This is about the amount of money that a family can spend after taxes (disposable household income)

^f^How would you place yourself on a line from 1 to 7, where 1 means that the measures against climate change have gone too far and 7 that the measures should go further?

^g^How convinced are you that climate change is mainly caused by human activity? Please place yourself on a scale from 0 to 100% where 0% means that you think it is extremely unlikely that climate change is caused mainly by human activity, 100% means that you are sure that climate change is caused mainly by human activity, and 50% means that you are unsure whether or not climate change is caused mainly by human activity

## Footnotes

1 In the Dutch case, making this claim is hampered by a scarcity of longitudinal measures of affective polarization, but indirect measures and proxies suggest that affective polarization increased over the past two decades (after a period of affective depolarization), but that the situation stabilized in more recent years.
